# The Ebola-effect in Guinea 2014-15: Tangled trends of malaria care in children under-five

**DOI:** 10.1371/journal.pone.0192798

**Published:** 2018-02-28

**Authors:** Delphin Kolie, Bienvenu S. Camara, Alexandre Delamou, Abdoul H. Béavogui, Veerle Hermans, Jeffrey K. Edwards, Guido Benedetti, Claude P. Muller, Johan van Griensven, Rony Zachariah

**Affiliations:** 1 Centre National de Formation et de Recherche en Santé Rurale de Maferinyah, Forécariah, Guinea; 2 Department of Public Health, Gamal University of Conakry, Conakry, Guinea; 3 Woman and Child Health Research Centre, Institute of Tropical Medicine, Antwerp, Belgium; 4 Médecins sans Frontières, Medical Department (Operational Research), Operational Centre Brussels, Luxembourg, Luxembourg; 5 University of Washington, Department of Global Health, Seattle, Washington, United States of America; 6 Department of Infection and Immunity, Luxembourg Institute of Health, Esch-sur-Alzette, Luxembourg; 7 Laboratoire National de Santé, Dudelange, Luxembourg; 8 Clinical Sciences Department, Institute of Tropical Medicine, Antwerp, Belgium; Centro de Pesquisas Rene Rachou, BRAZIL

## Abstract

**Introduction:**

The 2014–15 Ebola outbreak in West Africa was disruptive for the general health services in the affected countries. This study assessed the impact of the outbreak on the reported number and management of malaria in children under-five in rural Guinea.

**Materials and methods:**

A retrospective cross-sectional study was conducted in nineteen health centres in two rural, malaria-endemic health districts, one at the epicentre of the outbreak (Guéckédou) and one (Koubia) spared by Ebola. Routine surveillance data at health facility level were compared over similar periods of high malaria transmission in both districts before, during and after the outbreak.

**Results:**

There were significant declines in the number of visits during the Ebola outbreak (3,700) in Guéckédou compared to before (4,616) and after it (4,195), while this trend remained more stable within the three periods for Koubia. Differences were nonetheless significant in both districts (p<0.001). In 2014, during the peak of the outbreak, the overall number of malaria cases treated exceeded the number of confirmed malaria cases in Guéckédou. There were decreases in antimalarial treatment provision in August and November 2014. In contrast, during 2015 and 2016, the proportion of malaria positive cases and those treated were closely aligned. During the peak of the Ebola outbreak, there was a significant decrease in oral antimalarial drug administration, which corresponded to an increase in injectable antimalarial treatments. Stock-outs in rapid diagnostic tests were evident and prolonged in Guéckédou during the outbreak, while more limited in Koubia.

**Conclusion:**

The Ebola outbreak of 2014–15 in Guinea had a significant impact on the admission and management of malaria in children under-five. This study identifies potential challenges in the delivery of care for those at highest risk for malaria mortality during an Ebola outbreak and the need to improve preparedness strategies pre-Ebola and health systems recovery post-Ebola.

## Introduction

Children in Africa are frequently exposed to malaria, with one child dying of malaria every two minutes. In 2015, the African continent carried 66% (292,000/438,000) of the global under-five malaria-attributable deaths. In 2014, Guinea, a country with a high malaria burden, was at the epicentre of the West-African Ebola outbreak, which lasted until 2015 and was declared an international public health emergency [1–3]. The sustained nature of the Ebola outbreak jeopardized the health system in Guinea and its neighbouring afflicted countries, Sierra-Leone and Liberia. In these countries, many health facilities were shut down due to the lack of protective measures for health workers, Ebola-related death of health workers and the overall fear of contracting Ebola. Declines in maternal, paediatric and malaria related consultations and attrition from HIV/AIDS care have been reported in Guinea and Sierra Leone [4–10].

The Ebola outbreak represented a unique challenge to malaria control because both malaria and Ebola cases frequently present with fever as the primary complaint. As such, this “symptom overlap” may deter individuals with suspected malaria (or their care givers) from presenting to health facilities for fear of being labelled an Ebola case [11]. The correct diagnosis and treatment of children under five years of age is of importance because they represent a subpopulation at highest risk of mortality from both malaria and Ebola [1, 12, 13].

The Guinean National Malaria Control Programme (NMCP) states that all children with fever should be tested for malaria by a rapid diagnostic test (RDT) or microscopy, before being placed on treatment. Treatment for malaria includes artemisinin-based combination oral therapies (ACTs) for uncomplicated malaria and parenteral antimalarial drugs for severe malaria. Malaria diagnostic tests and treatment of severe malaria require the use of needles to collect finger prick blood or to inject an antimalarial medication. During the Ebola outbreak, these procedures conflicted with the overall recommendation to avoid invasive procedures and the “No Touch” policy, which was aimed at limiting Ebola transmission [14,15].

We hypothesized that the Ebola outbreak may have affected health-facility attendance for fever as well as the availability and use of malaria diagnostic tests and antimalarial drugs. This situation may have been different between districts affected by Ebola and those not-affected within the same country. Findings could be vital to guide malaria control strategies and improve Ebola recovery efforts in the future.

The aim of this study was to measure and compare the reported number of malaria cases and their management in children under-five years of age during the high transmission period of malaria before, during and after the 2014–15 Ebola outbreak in two districts in rural Guinea, one affected (Guéckédou) and the other, unaffected (Koubia) by the Ebola outbreak.

## Materials and methods

### Study design

This was a retrospective, cross-sectional study using routine malaria surveillance data.

### General setting

Guinea lies in West Africa and is bordered in the south by Liberia and Sierra Leone, which were also affected by the 2014–15 Ebola outbreak. The country has approximately 11 million inhabitants with 17% of the population being under 5 years old. Malaria is prevalent throughout the year, but transmission is highest from July to October. Plasmodium falciparum accounts for 98% of malaria cases. Under-five mortality is estimated to be 123 per 1,000 live births. Malaria is a leading cause of under-fives morbidity in Guinea, particularly in rural areas, with a 53% malaria proportional morbidity compared to 18% in urban areas. Malaria management in under-fives is guided by the NMCP and includes preventive (seasonal chemotherapy), diagnosis (biological) and treatment packages. Since July 2015, ten rural districts, out of 33, have been covered by a seasonal chemotherapy programme [14,16].

Malaria management flowcharts are available at most health facilities and are regularly updated based upon World Health Organization (WHO) recommendations. However, the health system still faces challenges: according to the 2012 national demographic and health survey, only a limited proportion (<10%) of under-five children received appropriate ACT treatment. In addition, most of the hyper-endemic rural districts are not yet covered by the seasonal chemotherapy programme [14,16].

### Specific setting

The study was conducted in the rural health districts of Guéckédou and Koubia. Guéckédou was relevant to inform the study objectives as the epicentre of the 2014/2015 Ebola outbreak. Guéckédou recorded the highest Ebola mortality (76%) countrywide, and belonged to the natural region of the country with the highest malaria burden [16,17]. Guéckédou is located in the south-east of the country with approximately 300,000 inhabitants. It has one district hospital, one communal health centre, 13 health centres and 25 health posts. The first and last case of Ebola was reported in March 2014 and December 2014 respectively. Guéckédou health district reported a total of 382 confirmed and probable cases of which 76% died. In addition, nine healthcare workers, out of 13 affected, died. Guéckédou is among the malaria hyper-endemic areas in Guinea, but is not covered by the on-going seasonal chemotherapy programme [14,17,18].

Koubia was randomly selected from the list of Ebola-unaffected districts with higher malaria incidence (≥ 10%) countrywide [19,20]. It is located in the central part of the country with approximately 100,000 inhabitants [16]. It has one district hospital, six health centres and six health posts [16]. The Koubia health district is covered by a seasonal chemotherapy programme with 17,763 (2016) and 18,739 (2017) under-fives been reached by the two first campaigns [21].

### Malaria management in Guinea

Guinea follows WHO guidelines for malaria management. All individuals presenting with fever to a health facility are subjected to a malaria RDT or blood microscopy. Uncomplicated malaria cases receive oral ACTs and first-line treatment includes Artesunate-Amodiaquine and Artesunate-Lumefantrine. Severe malaria cases are given parenteral Artesunate, Arthemeter or Quinine. In order to improve community access to malaria treatment, trained community health workers (CHWs) perform RDT screening and offer oral first-line drug treatment. CHWs refer complicated malaria cases to their affiliated health posts or health centres [14].

### Study population and period

All under-five children presenting to the 13 health centres in Guéckédou and the 6 health centres in Koubia districts were included. The study spanned comparable periods before (July-December 2013), during (July-December 2014 and 2015) and after (July-December 2016) the Ebola outbreak.

### Data variables and sources of data

Data on malaria management at primary health level are gathered and reported to the district officer monthly. The district officer compiles it, together with malaria activities of the district hospital, and submits monthly malaria reports to the Guinean NMCP. Data quality checks are routinely implemented during the data collection process. Study variables on malaria were extracted from the monthly malaria reports at district level and variables included were numbers of clinical visits, malaria suspects, non-suspects, cases tested for malaria (either by RDT or microscopy) and outcome, cases treated for malaria stratified by oral and injectable antimalarial treatment, and days of RDT stock-outs. Data on Ebola were sourced from the national Ebola situation report issued by the Ministry of Health.

### Statistical analysis

Data were entered into a dedicated EpiData database (version 3.1 for entry EpiData Association, Odense, Denmark). Malaria cases and management indicators (overall visits, malaria suspect, non-suspect, tested, positive and treated cases) were measured as monthly counts or median number per month. Only data from the highest malaria transmission period (July-December) of the year were considered for analysis. Counts were depicted in epidemiological curves before, during and after the Ebola outbreak in the affected (Guéckédou) and non-affected (Koubia) districts. Differences in yearly proportions (before, during and after the outbreak) by district were assessed using Pearson’s Chi-square test. The level of significance was set at a two-tailed p-value < 0.05.

### Ethics approval

Permission to carry out this study was obtained from the National Ethics Committee in Heath Research of Guinea, Conakry, Guinea. The National Ethics Committee in Health Research of Guinea approved the present study prior to any data collection. Data are fully anonymised as an aggregated malaria monthly report at district level. Only these aggregated data were used in the study, hence patient confidentiality was respected at all time and patient consent was not needed for the data collection. This research fulfilled the exemption criteria set by the Médecins Sans Frontières Ethics Review Board for a posteriori analyses of routinely collected clinical data and thus did not require MSF ERB review. It was conducted with permission from the Medical Director, Operational Centre Brussels, Médecins Sans Frontières.

## Results

**Fig 1:**
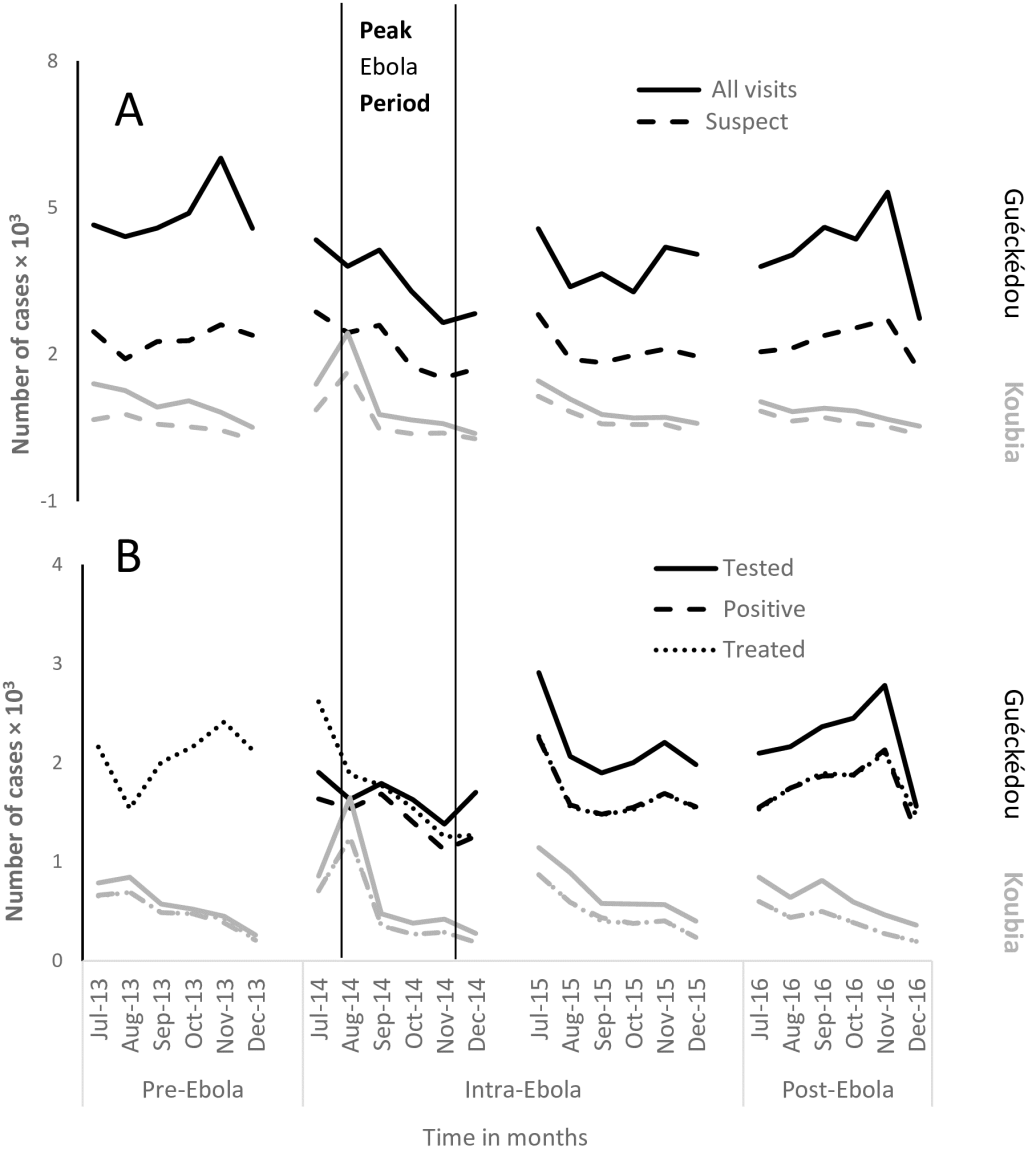
Monthly trends of visits, malaria cases and their management in under-fives, Guinea. (A): numbers of all visits and malaria suspect cases in under five children at the health centres in the health districts of Guéckédou and Koubia before (2013), during (2014–2015) and after (2016) the Ebola outbreak in Guinea; (B): numbers of cases tested for malaria, malaria positive, and treated for malaria in under five children at health centres in the health districts of Guéckédou and Koubia before (2013), during (2014–2015) and after (2016) the Ebola outbreak in Guinea.

The monthly trends of visits and malaria suspect cases among under-five children at the health centres of Guéckédou and Koubia are shown in Fig 1(A). There were significant declines in the number of visits during the Ebola outbreak in Guéckédou compared to before and after it, while this trend remained more stable within the three periods for Koubia. The reported monthly median number of visits in Guéckédou was 4,616 before the outbreak (2013), then had the greatest decline in patient visits during September-November 2014 with a median number of visits of 3,298 per month (a median number of visits of 3,700 per month in 2014). Afterwards, there was a progressive increase in the number of visits in reaching a peak of 5,317 in November 2016 (a median number of visits of 4,195 per month in 2016). There were significant differences in the proportions of malaria suspect cases among under-five children attending the health centres across the study period, see Table 1, p<0.001.

**Table 1:** Malaria suspect cases among under-five children attending health centres in the health districts of Guéckédou and Koubia in Guinea from 2013–2016.

Districts/Variables		2013		2014		2015		2016		P-value
		Number	*%*	Number	*%*	Number	*%*	Number	*%*	
*Guéckédou*	*Suspects*	13,814	*48*	12,697	*60*	12,226	*53*	13,287	*54*	< 0.001
	*Non- suspects*	15,034	*52*	8,367	*40*	10,898	*47*	11,394	*46*	
*Koubia*	*Suspects*	3,255	*54*	4,037	*65*	4,084	*77*	3,668	*76*	< 0.001
	*Non- suspects*	2,712	*46*	2,195	*35*	1,241	*23*	1,140	*24*	
Chi-square test										

The monthly trends of patients tested, positive results and malaria cases treated among under-five children are shown in Fig 1(B). In 2014, during the peak of the outbreak, with the Guéckédou health district, the overall number (10,333) of malaria cases treated exceeded the number of confirmed (8,639) malaria cases. There were decreases in antimalarial provision in 2014 during August and November in Guéckédou, which did not occur to the same degree in Koubia.

**Fig 2:**
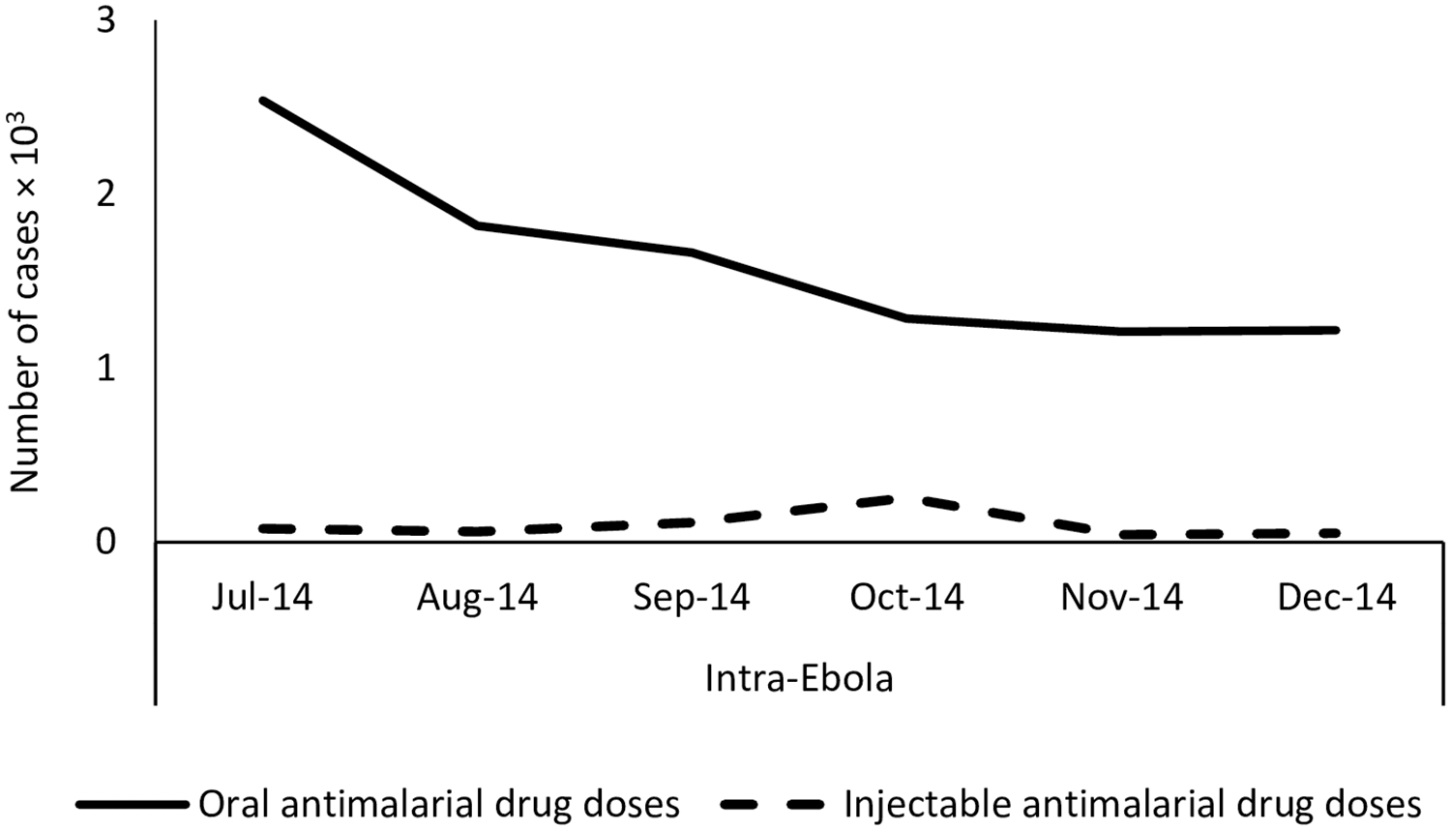
Monthly trends in numbers of oral/injectable antimalarial drug doses administered to under-fives in 2014, Guinea.

**Fig 3:**
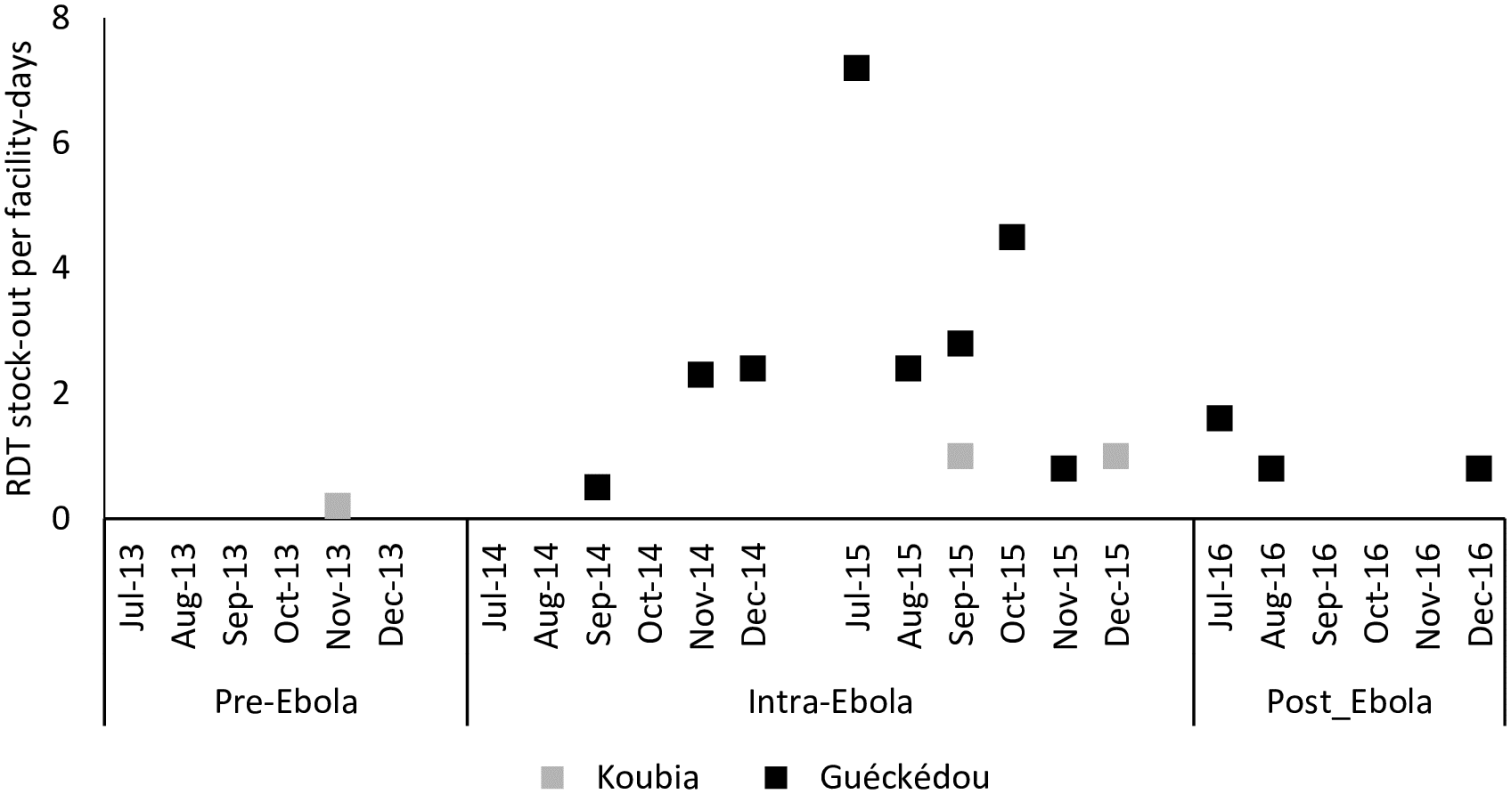
Number of monthly RDT stock-out facility-days in 2013–2016 in Guinea.

Reported oral and injectable antimalarial drug doses administered to under-five children at health centres remained stable through the study period in both districts except for Guéckédou in 2014. There was a clear decrease in oral antimalarial drug administration during October 2014, which contrasted with an increase in injectable antimalarial treatment administration: the proportion of injectable drug doses out of the total drug doses administered was 6% in September, 17% October and 3% in November 2014, respectively (see Fig 2, p<0.001). The health district of Guéckédou experienced 11 malaria RDT stock-outs across the period during and after the Ebola outbreak while they were 3 in Koubia, (Fig 3).

## Discussion

To our knowledge, this is the first study to describe and compare malaria burden and case management in children under-five, between an Ebola affected and non-affected district, in Guinea before, during and after the Ebola outbreak of 2014–15. Our findings show that in the Ebola-affected district of Guéckédou, there was a decrease in total clinical visits, malaria testing, reported malaria cases and increase in unconfirmed malaria case treatment during the Ebola outbreak. Additionally, there were more frequent RDT supply stock-outs. In contrast, there was a limited decline in under-five related health services attendance and stock-outs in the Ebola non-affected district (Koubia).

As described in other studies looking at the effects of the Ebola outbreak on healthcare systems, we found a dramatic decrease during the peak months in the counts of clinical visits for children under-five, to the reporting health facilities in Guéckédou [6,8,9]. This decrease in reported healthcare system usage could have been related to several factors. It is possible that people visited the health facilities less frequently because of concern about contracting Ebola there. Healthcare facilities in this Ebola epidemic have been associated with increased transmission rates of the virus [13,19]. This is of particular concern for those under-five, because they were one of the subgroups with the highest Ebola-related mortality rates [12].

Another plausible explanation for the decrease in clinical visits reported in Guéckédou could be related to the concern about being diagnosed with Ebola incorrectly. Both Ebola and malaria typically present with fever. During the initial screening process for possible Ebola-suspect cases, patients with malaria could meet the clinical case definition, which included fever≥ 38.0°^C^ plus three other associated symptoms (fatigue, muscle/joint pain, headache, loss of appetite, nausea and vomiting, stomach pain) [17]. Patients matching the definition for a suspect Ebola case were isolated, ideally transferred to an Ebola treatment centre, while waiting for the return of their Ebola screening test results. It remains unknown how many people may have been placed in suspect areas without Ebola and discharged with negative results, only to develop Ebola in the weeks that followed. Children under-five, would be at highest risk for this form of transmission to occur [13].

Moreover, patients referred to the Ebola treatment centre (ETC) during the epidemic were systematically tested for malaria and treated if needed [13,22]. Though we could not access data on the number of patients tested and treated for malaria in the ETC, the number of under-five malaria cases managed there could explain the decreased seen in malaria cases at the routine health centres. A study in Donka ETC in Conakry reported 72% of malaria comorbidity among acute Ebola patients [22].

Lastly, during the Ebola outbreak, healthcare provider resources were significantly stretched. Healthcare workers in Ebola-affected areas frequently became infected or were concerned about being infected and subsequently less providers were available [23]. This decrease in healthcare worker supply could have led to reduced number of clinical visits being completed and reported—also for malaria—during the peak of the outbreak in Guéckédou.

However, a study conducted by Moisan et al suggested a modest decrease in patients of all ages visiting health facilities from 2013 to 2014 in Guéckédou [24]. The difference with our findings suggests that under-five children had less chance of utilizing health services than the general population during the Ebola outbreak. Qualitative investigations on care seeking behaviours during the outbreak could permit better understanding of this consistent decrease in facility attendance and contribute to improving post-Ebola access to health services for children.

In 2013, the Guinean NMCP guidelines recommended that only those with suspect malaria that had a positive diagnostic malaria test (RDT and/or microscopy) should be treated with an ACT. The goal was to limit the inappropriate diagnosis and over prescribing of antimalarial drug which leads to drug resistance [14]. Our study found that in the Ebola non-affected district of Koubia, these guidelines were being followed: the doses of ACT reported dispensing mirrored the reported number of positive malaria tests. However, during the Ebola epidemic, the opposite was true in Guéckédou: the number of those treated with ACTs was higher than those with positive malaria tests.

This deviation from the national malaria guidelines during the Ebola outbreak in Guéckédou could be related to recommendations to limit invasive testing in this region. It is possible that clinicians were more likely to treat suspect malaria patients, especially under-five children, with ACTs, without completing a confirmatory malaria test, implying the invasive prick of the finger. This is of critical importance—because if an under-five child presents with symptoms of malaria in an Ebola affected area and tests negative for malaria, their likelihood of having Ebola increases [13]. It remains unknown how many of these children actually had malaria, or worse, Ebola.

This study also found another unique change in service delivery for malaria during the peak of the Ebola outbreak in Guéckédou: the reported usage of oral ACTs decreased while that of parental therapy increased. During the months of September, October and November 2014, the number of reported oral ACT usage decreased, while the use of parental treatment increased from August to October, the peak of the outbreak. Such an increase in parenteral anti-malaria therapy was not observed at any other time during the study period in either Guéckédou or Koubia. It remains unclear as to why there was an increase in parenteral use especially with the halt on invasive procedures during the outbreak, but this could be related to limited supply of oral ACTs during the peak of the Ebola outbreak or children who at the time of presentation were in severe clinical state due to delays in presentation. Lastly, there were more and prolonged stock-outs of RDTs supplies reported in Guéckédou compared with Koubia, 11 facility stock-out days versus 3, respectively. This is not surprising given the stress on the healthcare system as a whole, while trying to respond to the Ebola outbreak [23]. The lack of RDTs in such emergency context could affect proper diagnosis of some febrile illnesses and lead to under-estimation of malaria cases.

Our study findings lead to several policy recommendations for the Guinean healthcare system going forward. First, it is crucial that with future Ebola preparedness planning, adequate resources and training is available to provide the necessary screening and malaria/Ebola differential diagnosis protocols among those children presenting to health facilities. Secondly, it is key to have sufficient supplies of antimalarial medications, realizing that during an Ebola outbreak, logistical support may be shifted away, when it is most needed during the peak of the malaria season. Finally, it remains unclear how many children under-five had concurrent malaria and Ebola, however, a rapid Ebola screening test, similar to what is used for malaria, is desperately needed to assist in management of our highest risk and youngest patients [13].

### Strengths and limitations

This study has some limitations related to data missing in the Ebola affected district of Guéckédou and the comparativeness of the health districts selected. First, we were unable to assess the number of cases tested (or results) and RDT stock-outs during 2013 in Guéckédou. Second, data were not available in three and four health centres from August to November and December 2014 respectively. Third, the size of the two districts selected for this study was different and this could have an impact on the differences observed. To address this and ensure a comparativeness of the two health districts, trends of study variables were only, where possible, compared within and between the districts. Finally, we could only include two districts in this study, thus the sample may not be representative of the national situation. A study strength was the inclusion of all primarily health facilities of two districts with a high burden of malaria. Additionally, our data covers the highest periods of malaria transmission in Guinea and allow comparison between an affected and non-affected district. Thus, these results might be relevant for districts with the same Ebola and malaria epidemiological characteristics in Guinea and elsewhere. Lastly, the implementation and reporting of this study also adhered to the internationally recognized STROBE guidelines [25].

## Conclusion

The Ebola outbreak of 2014–15 in Guinea likely had a significant impact on the diagnosis and management of malaria in children under-five. Total clinical visits and reported malaria confirmed cases were decreased in an Ebola-affected health district when compared to a non-affected. This study identifies potential challenges in the delivery of care for those at highest risk for malaria mortality during an Ebola outbreak and the need to improve preparedness strategies going forward.
